# The prevalence of metabolic syndrome and metabolically healthy obesity in Europe: a collaborative analysis of ten large cohort studies

**DOI:** 10.1186/1472-6823-14-9

**Published:** 2014-02-01

**Authors:** Jana V van Vliet-Ostaptchouk, Marja-Liisa Nuotio, Sandra N Slagter, Dany Doiron, Krista Fischer, Luisa Foco, Amadou Gaye, Martin Gögele, Margit Heier, Tero Hiekkalinna, Anni Joensuu, Christopher Newby, Chao Pang, Eemil Partinen, Eva Reischl, Christine Schwienbacher, Mari-Liis Tammesoo, Morris A Swertz, Paul Burton, Vincent Ferretti, Isabel Fortier, Lisette Giepmans, Jennifer R Harris, Hans L Hillege, Jostein Holmen, Antti Jula, Jenny E Kootstra-Ros, Kirsti Kvaløy, Turid Lingaas Holmen, Satu Männistö, Andres Metspalu, Kristian Midthjell, Madeleine J Murtagh, Annette Peters, Peter P Pramstaller, Timo Saaristo, Veikko Salomaa, Ronald P Stolk, Matti Uusitupa, Pim van der Harst, Melanie M van der Klauw, Melanie Waldenberger, Markus Perola, Bruce HR Wolffenbuttel

**Affiliations:** 1 Department of Endocrinology, University of Groningen, University Medical Center Groningen, HPC AA31, P.O. Box 30001, Groningen RB 9700, The Netherlands; 2 Institute for Molecular Medicine Finland (FIMM), University of Helsinki, Helsinki, Finland; 3 Unit of Public Health Genomics, National Institute for Health and Welfare, Helsinki, Finland; 4 Research Institute of the McGill University of Health Centre, Montreal, Canada; 5 University of Tartu, Estonian Genome Center, Tartu, Estonia; 6 Center for Biomedicine, European Academy of Bolzano/Bozen (EURAC), Bolzano, Italy; 7 Data to Knowledge Research Group, School of Social and Community Medicine, University of Bristol, Bristol, UK; 8 Institute of Epidemiology II, Helmholtz Zentrum München, German Research Center for Environmental Health, Neuherberg, Germany; 9 Department of Epidemiology, University of Groningen, University Medical Center Groningen, Groningen, The Netherlands; 10 Genomics Coordination Center, University of Groningen, Groningen Bioinformatics Center, and University Medical Center Groningen, Groningen, The Netherlands; 11 Research Unit of Molecular Epidemiology, Helmholtz Zentrum München, German Research Center for Environmental Health, Neuherberg, Germany; 12 Ontario Institute for Cancer Research, Toronto, ON, Canada; 13 Department of Genes and Environment, Division of Epidemiology, The Norwegian Institute of Public Health, Oslo, Norway; 14 Department of Cardiology, University of Groningen, University Medical Center Groningen, Groningen, The Netherlands; 15 HUNT Research Center, Department of Public Health and General Practice, Faculty of Medicine, Norwegian University of Science and Technology, Trondheim, Norway; 16 THL-National Institute for Health and Welfare, Helsinki, Finland; 17 Department of Laboratory Medicine, University of Groningen, University Medical Center Groningen, Groningen, The Netherlands; 18 Department of Chronic Disease Prevention, National Institute for Health and Welfare, Helsinki, Finland; 19 Department of Neurology, Central Hospital, Bolzano, Italy; 20 Department of Neurology, University of Lübeck, Lübeck, Germany; 21 Pirkanmaa hospital district and Finnish Diabetes Association, Tampere, Finland; 22 University of Groningen, University Medical Center Groningen, LifeLines Cohort Study, Groningen, The Netherlands; 23 Institute of Public Health and Clinical Nutrition, University of Eastern Finland, and Research Unit, Kuopio University Hospital, Kuopio, Finland

**Keywords:** Harmonization, Obesity, Metabolic syndrome, Cardiovascular disease, Metabolically healthy

## Abstract

**Background:**

Not all obese subjects have an adverse metabolic profile predisposing them to developing type 2 diabetes or cardiovascular disease. The BioSHaRE-EU Healthy Obese Project aims to gain insights into the consequences of (healthy) obesity using data on risk factors and phenotypes across several large-scale cohort studies. Aim of this study was to describe the prevalence of obesity, metabolic syndrome (MetS) and metabolically healthy obesity (MHO) in ten participating studies.

**Methods:**

Ten different cohorts in seven countries were combined, using data transformed into a harmonized format. All participants were of European origin, with age 18–80 years. They had participated in a clinical examination for anthropometric and blood pressure measurements. Blood samples had been drawn for analysis of lipids and glucose. Presence of MetS was assessed in those with obesity (BMI ≥ 30 kg/m^2^) based on the 2001 NCEP ATP III criteria, as well as an adapted set of less strict criteria. MHO was defined as obesity, having none of the MetS components, and no previous diagnosis of cardiovascular disease.

**Results:**

Data for 163,517 individuals were available; 17% were obese (11,465 men and 16,612 women). The prevalence of obesity varied from 11.6% in the Italian CHRIS cohort to 26.3% in the German KORA cohort. The age-standardized percentage of obese subjects with MetS ranged in women from 24% in CHRIS to 65% in the Finnish Health2000 cohort, and in men from 43% in CHRIS to 78% in the Finnish DILGOM cohort, with elevated blood pressure the most frequently occurring factor contributing to the prevalence of the metabolic syndrome. The age-standardized prevalence of MHO varied in women from 7% in Health2000 to 28% in NCDS, and in men from 2% in DILGOM to 19% in CHRIS. MHO was more prevalent in women than in men, and decreased with age in both sexes.

**Conclusions:**

Through a rigorous harmonization process, the BioSHaRE-EU consortium was able to compare key characteristics defining the metabolically healthy obese phenotype across ten cohort studies. There is considerable variability in the prevalence of healthy obesity across the different European populations studied, even when unified criteria were used to classify this phenotype.

## Background

The current obesity epidemic is one of the greatest public health concerns of our century [1]. In Europe, obesity has reached epidemic proportions [2]. A study assessing data collected between 1997 and 2003 reported that the prevalence of obesity, defined as body mass index (BMI) ≥ 30 kg/m^2^, varied between 6% and 20%, with higher prevalence in Central and Eastern European countries and lower values in France, Italy, and some Scandinavian countries [3]. Among U.S. adults, obesity (BMI ≥ 30) prevalence has increased from 15% in the early 1970s to the most recent estimate of 34% in 2009–2010 [4,5]. Similar patterns are seen in other countries and were shown to be comparable across different age, ethnic, educational and income groups [6]. If the observed trends of increasing prevalence of obesity persist, by 2030 the absolute number of obese individuals could rise to a total of 1.12 billion, accounting for 20% of the world’s adult population [7].

Obesity is a major contributor to the global burden of chronic diseases and disabilities [1]. Increased adiposity is a key risk factor for type 2 diabetes, dyslipidaemia and cardiovascular disease, and is associated with many other conditions, including osteoarthritis, certain types of cancer, mental health, and increased mortality [8-13]. However, recent evidence indicates that obesity does not always lead to adverse metabolic effects such as impaired glucose tolerance, insulin resistance, dyslipidaemia and hypertension [14], a cluster of the obesity-driven alterations also known as the metabolic syndrome (MetS) [15,16]. A subgroup of approximately 10-30% of obese individuals is metabolically healthy despite having excessive accumulation of body fat [17-22]. This phenomenon is referred to in the current literature as metabolically healthy obesity (MHO) [23]. However, to date, little is known about the factors that delay onset of or protect obese individuals from developing metabolic disturbances [24].

Accumulating evidence indicates that the prevalence of MHO varies considerably based on the set of criteria used for its classification as well as on the cut-off values for each parameter included [19,24,25]. In addition, other factors such as lifestyle, ethnicity, sex, or age can largely influence the prevalence of MHO [19]. Recent observational studies show that the MHO phenotype is associated with lower risk of CVD [26] and mortality, especially in those physically active [27], although not all studies could confirm these findings [28]. This highlights the importance of investigating MHO using harmonized classification criteria and studying the extent to which MHO is associated with the risk for chronic diseases.

The BioSHaRE-EU Project is an international collaborative project between European and Canadian Institutes and European cohort studies. It aims to harmonize data from clinical examinations and analytical results from biospecimens, as well as measures of life style, social circumstances and environmental exposures. Computing infrastructure is developed enabling the effective pooling of data and research into critical sub-components of the phenotypes associated with common complex diseases (http://www.bioshare.eu) [29-31]. The Healthy Obese Project (HOP) is the first scientific project in BioSHaRE to use these tools in order to gain insights into the characterization, the determinants and consequences of (healthy) obesity. We report the results of the first phase of the HOP project, in which we jointly analysed data from 163,517 individuals in ten population-based cohort studies across Europe. The objectives were to assess the potential for harmonization and collaboration, and to evaluate the prevalence of MetS in obese participants using different classification criteria and by characterizing the clinical and metabolic factors associated with MHO.

## Methods

### Study participants

This study included participants from ten population-based cohort studies in seven European countries as listed below. Data from 163,517 individuals were available from the following cohort studies: Estonia: the population-based biobank of the Estonian Genome Project of University of Tartu (EGCUT) (n = 8,930) [32]; Finland: FINRISK2007 (DILGOM) (n = 3,685) [33] and Health2000 (H2000) (n = 6,022) [34]; Germany: the Cooperative Health Research in the Region of Augsburg (KORA) study (n = 2,987) [35], Italy: Collaborative Health Research in South Tyrol Study (CHRIS) (n = 1,117) and the MICROS study (n = 1,060) [36]; the Netherlands: LifeLines (n = 63,995) [37], and the Prevention of REnal and Vascular ENd stage Disease study (PREVEND) (n = 7,216) [38]; Norway: the Nord-Trøndelag health study (HUNT2 survey) (n = 61,199) [39]; and United Kingdom: the National Child Development Study birth cohort (NCDS), also known as the 1958 birth cohort (n = 7,306) [40]. A brief description of all participating studies is given in the Additional file 1: Study descriptions and methodologies.

All study participants were of European origin, aged between 18 and 80 years, and had participated in a clinical examination for anthropometric and blood pressure measurements. Blood samples were taken for analysis of lipids and glucose (Additional file 1: Study descriptions and methodologies). Participants were only included if all data on clinical and metabolic measurements needed to define the status of MetS and obesity were available,. All cohorts had gained approval through their local research ethics committees or institutional review board for secondary usage of data. Participants gave their written informed consent to their study of origin. The current study protocol also gained approval under the data access and ethics governance requirements of the study of origin. The data on the outcomes measured in this study have not been published before by the individual cohorts.

### Data harmonization process

Characteristics describing each cohort study (e.g. design, sample size) are catalogued in a systematic way on the BioSHaRE website (http://www.bioshare.eu). BioSHaRE investigators met at a workshop in order to define the set of variables to be generated from the harmonization process. These ‘target’ variables determine the data information content that is required from each study to generate compatible (i.e. harmonized) variables. By evaluating study-specific questionnaires, standard operating procedures and data dictionaries, used by the participating cohort studies, the potential for each cohort study to generate the target variables was determined. Then researchers working with the data transformed their data locally into a common harmonized format. Parts of this process have been published recently [31], and details related to pairing decisions taken and processing algorithms are available online (https://www.bioshare.eu/content/healthy-obese-project-dataschema).

### Classification of obesity, metabolic syndrome and the MHO phenotype

The criteria applied for measures of weight and height required that each cohort study measured participants when dressed in lightweight clothing and no shoes. Body mass index (BMI) was calculated as weight in kilograms divided by height in meters squared. Obesity was defined according to the current World Health Organization (WHO) classification as having a BMI ≥ 30 kg/m^2^[41].

Four clinical measures were used to define the MetS phenotype in the obese subjects based on the original NCEP ATP III definition [42]: 1) elevated blood pressure, defined as systolic blood pressure (SBP) ≥130 mmHg or diastolic blood pressure (DBP) ≥85 mmHg, or antihypertensive drug treatment; 2) elevated fasting blood glucose level ≥6.1 mmol/l or use of blood glucose lowering agents or history/diagnosis of type 2 diabetes; 3) decreased HDL-cholesterol level (<1.03 mmol/l in men or <1.30 mmol/l in women) or drug treatment aimed to increase HDL-cholesterol; and 4) hypertriglyceridaemia (triglyceride level ≥ 1.70 mmol/l) or drug treatment for elevated triglycerides (Table 1). Data on waist circumference was not available in all cohorts. However, > 95% of LifeLines participants with obesity had increased waist circumference according to the NCEP ATP III definition [42], and we therefore considered the presence of ≥ 2 of the four clinical measures as diagnostic for MetS [15]. In addition, we also applied a set of less strict criteria in which the cut-off levels for elevated systolic and diastolic blood pressure were set at ≥140 mmHg and ≥90 mmHg, respectively, and the cut-off level for elevated fasting blood glucose was set at 7.0 mmol/l. As the components of MetS can be influenced by smoking, we recorded whether the participants were current smokers.

**Table 1:** Criteria and the thresholds used for the definition of metabolically healthy obese individuals in each cohort study

	**Strict criteria**	**Less strict criteria**
Blood pressure	SBP ≥ 130 mmHg or DBP ≥ 85 mmHg or use of antihypertensive medication	SBP ≥ 140 mmHg or DBP ≥ 90 mmHg or use of antihypertensive medication
Elevated blood glucose	fasting blood glucose ≥ 6.1 mmol/l or non-fasting blood glucose ≥ 7.0 mmol/l or use of blood glucose lowering medication or diagnosis of type 2 diabetes	fasting blood glucose ≥ 7.0 mmol/l or non-fasting blood glucose ≥ 7.8 mmol/l or use of blood glucose lowering medication or diagnosis of type 2 diabetes
Decreased HDL-cholesterol	< 1.03 mmol/l in men or < 1.30 mmol/l in women or medical treatment for low HDL	< 1.03 mmol/l in men or < 1.30 mmol/l in women or medical treatment for low HDL
Elevated triglycerides*	≥ 1.70 mmol/l or medication for elevated triglycerides	≥ 1.70 mmol/l or medication for elevated triglycerides
Diagnosis for CVD	Yes	Yes

*Abbreviations*: *CVD* cardiovascular disease, *DBP* diastolic blood pressure, *SBP* systolic blood pressure.

The presence of ≥ 2 abnormal clinical measures (blood pressure, blood glucose, HDL-cholesterol, triglycerides) according to the strict criteria was considered diagnostic for MetS.

Metabolically healthy obesity is defined as having BMI ≥ 30, none of the following criteria of the metabolic syndrome [15,42], and no cardiovascular disease.

*In case of non-fasting measurements, the cut-off value was set at 2.10 mmol/l.

The methodology for measurement of the laboratory variables in the various studies is described in the Additional file 1. As not all participating cohorts had performed measurement of triglycerides in fasting serum samples, we corrected, as part of the harmonization process, non-fasting triglycerides values based on the findings of a recent report on the associations between fasting time and serum triglycerides levels (i.e. the threshold of 2.1 mmol/l was used) [43]. For the same reason, we used a different cut-off value for non-fasting blood glucose (i.e. thresholds of 7.0 mmol/l and 7.8 mmol/l for ‘strict’ and ‘less strict’ criteria were used, respectively (Table 1)). In the NCDS study, fasting blood glucose was calculated from HbA1c based on a regression formula obtained in the LifeLines Cohort Study (see Additional file 1).

We collected and analysed three types of information: (1) the presence of individual components of MetS in obese participants in each cohort study; (2) the number and percentage of MetS criteria fulfilled in obese participants in each cohort; and (3) the number and percentage of subjects fulfilling the criteria for being metabolically healthy obese in different age groups. MHO was established when subjects with obesity had none of the MetS components, and had no previous diagnosis of cardiovascular disease. As there were age differences between the cohorts, we performed age standardization against the European population, as defined by the EU-27 Member States population on January 1, 2010 (http://epp.eurostat.ec.europa.eu/portal/page/portal/statistics/search_database, accessed October 17, 2013). Prevalence was calculated for men and women separately based on 10-year age groups. The definition of prevalent cardiovascular disease varied slightly between cohorts (Additional file 1: The definition of cardiovascular disease), but in the majority of cohort studies, it was based on self-reported history of acute myocardial infarction, stroke, angina pectoris or cardiovascular intervention (CABG or PTCA).

### Statistical analyses

Results are presented as means ± standard deviation, or number and percentage. Frequency of individual components of MetS were calculated, both for the whole population of obese individuals and for specific age categories. If needed, data are given for men and women separately. As this is a descriptive observational study, no formal statistical testing was performed.

## Results

**Figure 1:**
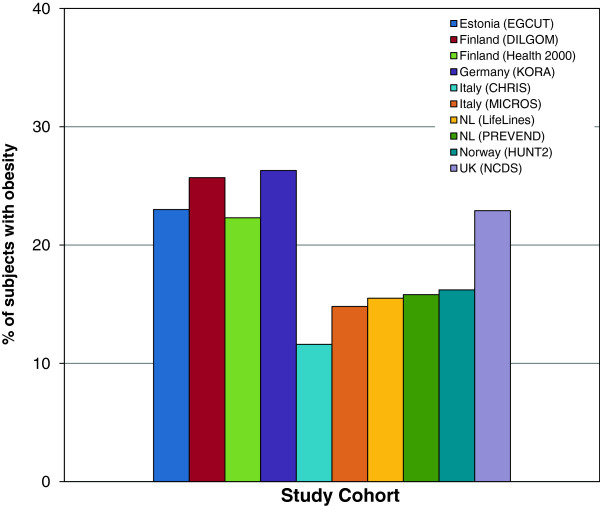
The prevalence of obesity in the participating cohorts given as a percentage of the total sample size of the cohort.

Overall, data for 163,517 individuals were available for the analysis, of whom 28,077 (17.2%) were obese (11,465 (15.8%) men and 16,612 (18.3%) women). Table 2 summarizes the clinical characteristics of obese participants from each cohort study. Mean age of the obese participants varied from 44.0 to 59.6 years. In all cohorts, the frequency of obesity was greater among women than among men (only statistically significant (P < 0.05) for Health2000, LifeLines, Prevend and HUNT2), while it was greater among men in the NCDS cohort (P = 0.033). The highest prevalence of obesity was found in Germany (26.3%, mean age of the participants 59.6 years), Finland (DILGOM cohort, 25.7%, 57.3 years), Estonia (23%, 52.6 years), and the United Kingdom (22.9%, 44.0 years), while the lowest prevalence of obesity was observed in the Italian studies CHRIS (11.6%, 53.6 years) and MICROS (14.8%, 54.9 years) (Figure 1). The percentage of individuals currently smoking varied between 15 and 31% (Table 2).

**Table 2:** Characteristics of the obese (BMI ≥ 30) participants

**Country & study**	**Estonia**	**Finland**		**Germany**	**Italy**		**The Netherlands**		**Norway**	**UK**
	**EGCUT**	**DILGOM**	**HeaIth2000**	**KORA**	**CHRIS**	**MICROS**	**LifeLines**	**PREVEND**	**HUNT2**	**NCDS**
Total number of participants (N)	8,930	3,685	6,022	2,987	1,117	1,060	63,995	7,216	61,199	7,306
Number with BMI ≥ 30 (%)	2,053 (23.0)	946 (25.7)	1,342 (22.3)	786 (26.3)	130 (11.6)	157 (14.8)	9,934 (15.5)	1,137 (15.8)	9,922 (16.2)	1,670 (22.9)
Gender (M (%)/F)	698 (34.0)/1,355	399 (42.2)/547	573 (42.7)/769	373 (47.5)/413	60 (46.2)/70	57 (36.3)/100	3,813 (38.4)/6,121	514 (45.2)/623	4,104 (41.4)/5,818	874 (52.3)/796
Age (yrs)	52.6 ± 14.1	57.3 ± 11.6	54.5 ± 12.8	59.6 ± 12.0	53.6 ± 12.9	54.9 ± 15.2	47.4 ± 11.7	53.5 ± 11.7	53.5 ± 15.4	44.0 ± 0
BMI (kg/m^2^)	34.4 ± 4.1	34.2 ± 4.1	33.6 ± 3.4	33.8 ± 3.7	33.1 ± 3.4	33.6 ± 4.5	33.6 ± 3.6	33.2 ± 3.3	33.2 ± 3.1	33.9 ± 3.8
Waist circumference (cm)	107 ± 12	110 ± 11	108 ± 10	109 ± 11	NA	NA	108 ± 10	105 ± 11	101 ± 10	106 ± 10
HDL cholesterol (mmol/l)	1.52 ± 0.33	1.30 ± 0.33	1.17 ± 0.32	1.31 ± 0.31	1.54 ± 0.45	1.54 ± 0.34	1.28 ± 0.33	1.16 ± 0.34	1.24 ± 0.35	1.38 ± 0.32
Men	1.35 ± 0.28	1.15 ± 0.26	1.05 ± 0.27	1.21 ± 0.29	1.31 ± 0.32	1.36 ± 0.25	1.13 ± 0.26	1.01 ± 0.27	1.10 ± 0.29	1.30 ± 0.30
Women	1.60 ± 0.32	1.42 ± 0.33	1.26 ± 0.32	1.40 ± 0.30	1.74 ± 0.45	1.65 ± 0.34	1.38 ± 0.33	1.29 ± 0.33	1.35 ± 0.36	1.47 ± 0.31
Triglycerides (mmol/I)	2.10 ± 1.16	1.82 ± 1.01	2.02 ± 1.22	1.77 ± 1.09	1.53 ± 0.99	1.87 ± 1.27	1.54 ± 1.02	1.88 ± 1.33	2.35 ± 1.39	2.17 ± 1.63
Blood glucose (mmol/I)	4.8 ± 1.8	6.4 ± 1.3	5.9 ± 1.7	5.9 ± 1.2	5.6 ± 0.9	5.4 ± 1.5	5.4 ± 1.3	5.4 ± 1.6	5.9 ± 2.0	4.9 ± 1.1
Systolic blood pressure (mmHg)	136 ± 17	140 ± 19	142 ± 20	128 ± 18	128 ± 14	143 ± 22	133 ± 15	139 ± 20	146 ± 22	132 ± 16
Diastolic blood pressure (mmHg)	84 ± 11	83 ± 11	87 ± 10	78 ± 10	83 ± 8	85 ± 11	77 ± 9	77 ± 10	85 ± 13	83 ± 10
Current smoking (%)	30.5	15.3	23.0	17.7	15.4	28.0	19.9	26.3	30.8	23.9
Number with MetS (M/F)	410/606	323/355	425/515	229/216	26/26	34/33	2,208/2,262	346/335	2,792/3,114	513/269
Number with MHO (M/F)	34/166	7/37	19/43	34/61	11/12	4/9	359/1,433	26/94	180/553	79/226

**Figure 2:**
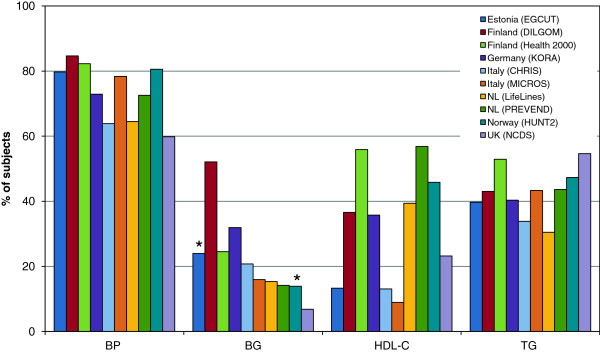
**The frequency of individual components of the metabolic syndrome among obese subjects (BMI ≥ 30 kg/m**^**2**^**).** The presence of the metabolic syndrome mainly depends on the presence of a high blood pressure followed by the level of triglycerides and HDL cholesterol and – to a lesser extent – blood glucose levels. BP = blood pressure, BG = blood glucose, HDL-C = high density lipoprotein cholesterol, TG = triglycerides. *Denotes non-fasting measurement of blood glucose.

**Figure 3:**
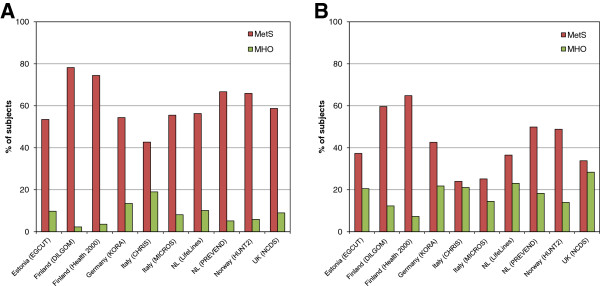
**Age-standardized prevalence of metabolic syndrome (MetS) and metabolically healthy obesity (MHO) amongst obese (BMI ≥ 30 kg/m**^**2**^**) individuals in the participating cohorts, separately shown for men (panel A, left) and women (panel B, right).**

The observed prevalence of MetS was mainly driven by the presence of elevated blood pressure with a range from 60% to 85% of individuals fulfilling the criterion for high BP (Table 3, Figure 2). In contrast, elevated blood glucose contributed least to MetS, although we did observe considerable diversity between the cohorts. The percentage of obese individuals with elevated blood glucose varied from 7% in the UK NCDS cohort to 52% in the Finnish DILGOM cohort. A similar difference was observed in the percentage of the obese individuals with decreased HDL-cholesterol level: the lowest prevalence was observed in the Italian studies (9% and 13% in the MICROS and CHRIS cohorts, respectively), while the highest prevalence was detected in the Dutch PREVEND cohort (57%). The percentage of the individuals with elevated triglyceride levels ranged between 31% in the Dutch LifeLines study and 55% in the UK NCDS participants. As a result, the age-standardized percentage of men with MetS according to the classic 2001 NCEP ATP III criteria ranged from 42.7% in the Italian CHRIS cohort to 78.2% in the Finnish DILGOM cohort, and for women from 24% in CHRIS to 64.8% in the Finnish Health2000 cohort (Figure 3A,B).

**Table 3:** The frequency of individual components of the metabolic syndrome in obese (BMI ≥ 30) individuals

	**Estonia**	**Finland**		**Germany**	**Italy**		**The Netherlands**		**Norway**	**UK**
	**EGCUT**	**DILGOM**	**Health2000**	**KORA**	**CHRIS**	**MICROS**	**LifeLines**	**PREVEND**	**HUNT2**	**NCDS**
Total N	2,053	946	1,342	786	130	157	9,934	1,137	9,922	1,669
Metabolic component										
Strict criterium for high BP (%)	1,637 (79.7)	801 (84.7)	1,104 (82.3)	573 (72.9)	83 (63.9)	123 (78.3)	6,407 (64.5)	825 (72.6)	7,991 (80.5)	998 (59.8)
Strict criterium for blood glucose (%)	482 (23.4)	493 (52.1)	329 (24.5)	251 (31.9)	27 (20.8)	25 (15.9)	1,524 (15.3)	161 (14.2)	1,377 (13.9)	114 (6.8)
Criterium for HDL cholesterol (%)	273 (13.3)	346 (36.6)	750 (55.9)	281 (35.8)	17 (13.1)	14 (8.9)	3,913 (39.4)	646 (56.8)	4,547 (45.8)	387 (23.2)
Criterium for triglycerides (%)	815 (39.7)	407 (43.0)	710 (52.9)	317 (40.3)	44 (33.9)	68 (43.3)	3,028 (30.5)	496 (43.6)	4,693 (47.3)	912 (54.6)
Less strict criterium for high BP (%)	1,386 (67.5)	670 (70.8)	916 (68.3)	498 (63.4)	64 (49.2)	98 (62.4)	4,492 (45.2)	660 (58.2)	6,447 (65.0)	609 (36.5)
Less strict criterium for blood glucose (%)	463 (22.5)	176 (18.6)	148 (11.0)	135 (17.2)	12 (9.2)	12 (7.6)	825 (8.3)	87 (7.7)	920 (9.3)	92 (5.5)

As expected, when less strict MetS criteria were used, the percentage of obese individuals with elevated blood pressure or blood glucose was lower (Table 3). This also resulted in a lower number of subjects with MetS (Table 4A,B).

**Table 4:** Number of components of the metabolic syndrome (waist circumference not included) present among obese participants

**A. Number of MetS component (strict criteria)**	**Estonia**	**Finland**		**Germany**	**Italy**		**The Netherlands**		**Norway**	**UK**
	**EGCUT**	**DILGOM**	**Health2000**	**KORA**	**CHRIS**	**MICROS**	**Lifelines**	**PREVEND**	**HUNT2**	**NCDS**
Total N	2,053	946	1,342	786	130	157	9,934	1,137	9,922	1,669
0 criteria present (%)	242 (11.8)	46 (4.9)	76 (5.7)	98 (12.5)	38 (29.2)	18 (11.5)	1,808 (18.2)	120 (10.6)	755 (7.6)	305 (18.3)
1 criterium present (%)	793 (38.6)	222 (23.5)	326 (24.3)	243 (31.0)	40 (30.8)	72 (45.9)	3,656 (36.8)	336 (29.6)	3,261 (32.9)	582 (34.9)
2 criteria present (%)	689 (33.6)	309 (32.7)	400 (29.8)	227 (28.9)	31 (23.9)	45 (28.7)	2,604 (26.2)	323 (28.4)	2,916 (29.4)	565 (33.9)
3 criteria present (%)	270 (13.2)	269 (28.4)	393 (29.3)	147 (18.7)	15 (11.5)	20 (12.7)	1,456 (14.7)	286 (25.2)	2,445(24.6)	169 (10.1)
4 criteria present (%)	59 (2.9)	100 (10.6)	147 (11.0)	71 (9.0)	6 (4.6)	2 (1.3)	410 (4.1)	72 (6.3)	545 (5.5)	48 (2.9)
**B. Number of MetS components (less strict criteria)**	**Estonia**	**Finland**		**Germany**	**Italy**		**The Netherlands**		**Norway**	**UK**
	**EGCUT**	**DILGOM**	**Health2000**	**KORA**	**CHRIS**	**MICROS**	**LifeLines**	**PREVEND**	**HUNT2**	**NCDS**
Total N	2,053	946	1,342	786	130	157	9,934	1,137	9,922	1,669
0 criteria present (%)	381 (18.6)	112 (11.8)	134 (10.0)	140 (17.8)	53 (40.8)	29 (18.5)	2,767 (27.9)	172 (15.1)	1,335 (13.5)	452 (27.1)
1 criterium present (%)	778 (37.9)	319 (33.7)	375 (27.9)	260 (33.1)	35 (26.9)	77 (49.0)	3,582 (36.1)	344 (30.3)	3,304 (33.3)	613 (36.7)
2 criteria present (%)	619 (30.2)	307 (32.5)	426 (31.7)	221 (28.1)	27 (20.8)	39 (24.8)	2,290 (23.1)	355 (31.2)	2,901 (29.2)	456 (27.3)
3 criteria present (%)	227 (11.1)	166 (17.6)	331 (24.7)	131 (16.7)	12 (9.2)	11 (7.0)	1,084 (10.9)	229 (20.1)	2,027 (20.4)	117 (7.0)
4 criteria present (%)	48 (2.3)	42 (4.4)	76 (5.7)	34 (4.3)	3 (2.3)	1 (0.6)	211 (2.1)	37 (3.3)	355 (3.6)	31 (1.9)

Across all ten cohorts, a total of 3,387 obese participants (12%) did not have any metabolic abnormalities according to the strict definition of MetS, as well as no previous diagnosis of cardiovascular disease, as defined by the MHO phenotype. After age standardization, the highest prevalence of MHO in men was found in the Italian CHRIS study (19%) and in the German KORA study (13.5%), and in women in UK NCDS (28.4%), Dutch LifeLines (23.1%), KORA (21.8%) and CHRIS (21.1%). The lowest prevalence was observed in the two Finnish cohorts (2.3 and 3.6% for men, 7.3 and 12.3% for women) and the Norwegian HUNT2 study (5.9% in men, 14% in women) (Figure 3A,B).

The trend towards a higher percentage of MHO in women compared with men was evident in almost all studies. This sex difference was most apparent in the NCDS cohort, in which 28.4% of obese women were metabolically healthy in comparison with only 9% of obese men with the same phenotype (Figure 3). In contrast, the percentage of men and women with MHO was similar in the Italian CHRIS study (19% versus 21.1%). These findings were also independent of the definition of MHO, as we observed the same tendency with both strict and less strict criteria (data not shown).

**Figure 4:**
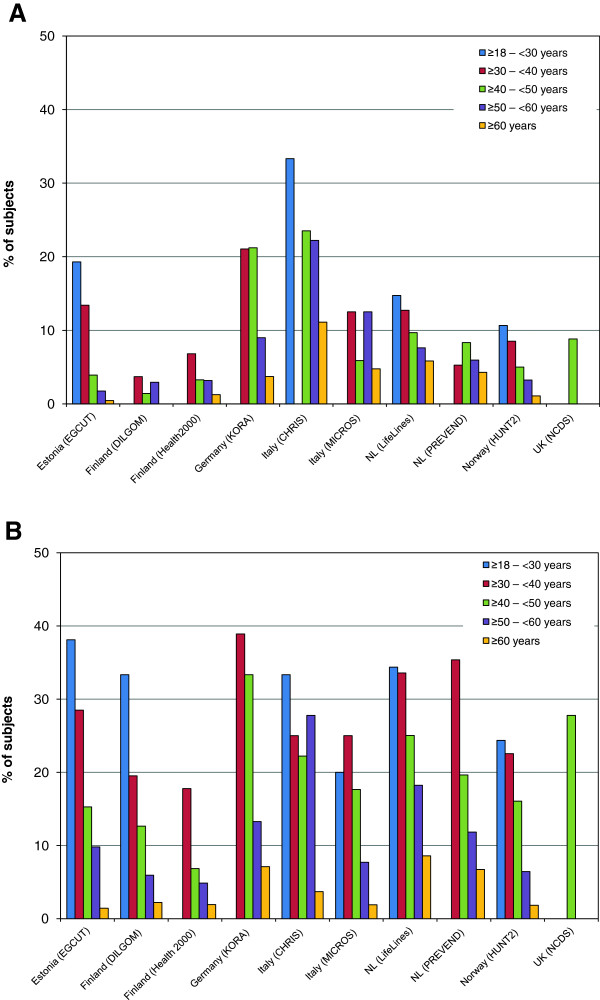
**Percentage of subjects (panel A: men; panel B: women) meeting the criteria of being ‘healthy obese’.** The results are stratified for different age groups. In general, within each cohort the prevalence of healthy obesity decreases with increasing age. Note that more females are metabolically healthier than males.

Overall, we observed a decrease in the prevalence of MHO with increasing age, independent of sex and the MetS definition criteria used (Figure 4A,B). This pattern was seen in all cohorts except the Italian CHRIS study, in which the prevalence of MHO appeared to be relatively constant until the age of 60. In all cohorts, a subset of the obese individuals remained metabolically healthy, even in the oldest age group (≥ 60 years). The highest prevalence of MHO among those 60 years and older was observed in the Dutch LifeLines study (8%).

## Discussion

In this large-scale collaborative study, we evaluated the prevalence of metabolic syndrome and healthy obesity among obese individuals using the data of 163,517 people from ten European cohort studies from seven different countries. We found considerable variation in the prevalence of both phenotypes suggesting that the distribution of the MetS and MHO across the different populations in general is not equal. However, our analysis did reveal a consistently higher prevalence of the MHO phenotype in women compared to men. Furthermore, the percentage of obese subjects with a favourable risk profile decreases with increasing age in all cohorts.

With the exception of the Italian, Norwegian and UK cohorts, the prevalence of obesity was much higher in the European populations we studied than was reported in the most recent review addressing the distribution of obesity in Europe [2]. Such differences may be due to potential underestimation of the prevalence of obesity in the systematic review because of the inclusion of studies using self-reported BMI [2]. In our study, the data on BMI were obtained through direct measurements made by trained research nurses or study assistants which provides more accurate estimation of obesity prevalence in the participating cohorts. Another explanation for the discrepancy in the prevalence patterns may be related to the difference in the time period when the studies were conducted. While the surveys included in the systematic review were performed between the mid-1980s and 2003, most of the data in our study were collected after 2000, with the earliest data available from 1995 and the most recent data from 2012. The differences in estimations of the obesity prevalence can, therefore, present different phases of an increasing trend. Although our data are obtained from large population-based cohort studies or biobanks, we have to realize that our results cannot always be generalized to the overall prevalence in the specific countries, as some cohorts have only collected data from a specific region of that country (CHRIS/MICROS/HUNT2), or from a specific age group (NCDS). Despite the detected variation, the data confirm the observations that obesity in European countries continued to rise the last decade and has reached epidemic proportions [2]. However, recent publications suggest levelling off of the obesity epidemic [44-46], although in subjects with lower socioeconomic status a steady increase in prevalence still is observed [47].

The Finnish cohorts had the highest prevalence of MetS among obese subjects and the lowest percentage of MHO. In contrast, in the Italian MICROS and the Dutch LifeLines studies we observed a lower prevalence of MetS among obese subjects together with a higher percentage of MHO. Similar patterns in the occurrence of MetS in Europe have been reported previously [48]. MetS is a constellation of metabolic risk factors, associated with an increased risk for the development of atherosclerotic cardiovascular disease as well as type 2 diabetes mellitus [15,16,49]. MetS has been shown to be the major risk determinant of heart disease, also when a population generally has low levels of HDL- and LDL-cholesterol [50]. The most frequent MetS component present in obese individuals was elevated blood pressure. In the 10 studies, obesity coincided with hypertension in 60% to 85% cases. In contrast, we observed considerable variations in the prevalence of other components of MetS, especially blood glucose and HDL-cholesterol. A blood pressure exceeding the strict criterion for a high blood pressure can be accounted as a main contributor promoting unhealthy obesity and metabolic syndrome in the Finnish cohorts in this study. Finnish tendency for elevated blood pressure has also been detected earlier, recently by The European Heart Network and The European Society of Cardiology [51].

Our study extends previous efforts to describe the phenomenon of healthy obesity and to estimate its prevalence in different countries in several important ways, including helping to disentangle whether differences in the prevalence of MHO are due to geographic variation or differences in measurements. Using a large amount of validated information, we applied a rigorous protocol to harmonize data from multiple population-based European studies, and ensure a high level of homogeneity of the MetS definition used to calculate the MHO prevalence. Recently, the lack of a standard approach to use the same sets of criteria and cut-off values to define metabolic abnormalities has been highlighted as the major source of the high variability in the reported MHO prevalence [19,24,25]. Yet, our results also demonstrate a significant diversity in the prevalence of MHO across Europe using the harmonized criteria to define MetS. The highest percentage of MHO in men was found in CHRIS and KORA, and in women in NCDS, LifeLines, KORA and CHRIS, whereas the lowest prevalence was found in the Finnish cohorts and in HUNT2. In our study, we have used the established risk factors associated with the metabolic syndrome [41,42] to identify the MHO phenotype. Our data on MetS components is consistent with the outcome of previously performed studies on the prevalence of the metabolic abnormalities in Europe [48,52]. As age and sex are important factors in the development of MetS, we have also evaluated the age- and sex-stratified prevalence of MHO per decade. Our results indicate a higher prevalence of the MHO phenotype in women than in men as well as an age-related decline in the percentage of obese subjects with a metabolically healthy phenotype [19,24]. Collectively, our findings raise additional questions about the underlying factors promoting the variation in the prevalence of MHO across different populations. Such variation in the distribution of metabolic phenotypes can be explained by several factors, including difference in age of the cohort participants, differences in environmental factors such as physical activity level, diet, smoking and alcohol use, and differences in the selection and inclusion of participants [52]. Also the psychosocial profile and genetic factors [19,24] may play a role. While behavioral factors, i.e. higher levels of physical activity or moderate alcohol intake, have been shown to be associated with the MHO phenotype [18], there is no evidence yet whether genetic background and divergence between populations does contribute to the metabolically favorable profile in obesity [24].

Given the number of serious health problems associated with obesity including type 2 diabetes, cardiovascular disease, and an increased risk for various types of cancer, the investigation of the healthy obesity phenotype may provide novel insights into the pathophysiology of obesity-related co-morbidities and help to identify at-risk obese individuals. Furthermore, it may help in the development of better interventions for obese patients. There are strong indications that weight loss may not have a beneficial effect on certain metabolic risk factors in MHO individuals [20] and even result in a paradoxical response [53]. Therefore, the one-size-fits-all approach regarding the consequences of obesity should be revisited, and the prevailing concept in the health care system that obesity is always bad should be re-evaluated. Also, a proper classification of the at-risk and metabolically benign obese individuals should be taken into account in medical research to prevent any bias in the interpretation of the results.

The main strengths of this descriptive study are the large sample size and the application of harmonized criteria to evaluate the prevalence of MetS and the degree of the MHO across different European cohort studies. Through our harmonization process [31], we have shown the possibility for collaborative research based on a careful harmonization process across multiple participating cohort studies. Several important factors may have a bearing on the results. First, we used BMI to define the obesity status. Since BMI is a measure of general obesity and cannot distinguish between fat and lean mass, other measures such as waist circumference (WC) or waist-hip-ratio (WHR) might be better indicators of visceral fat accumulation. Although a few studies reported lower fat accumulation in MHO individuals compared to the obese with metabolic abnormalities [17,24], no difference in the prevalence of MHO was found when WC was used instead of BMI to define the MHO phenotype in the NHANES cohort [18]. Second, although our harmonized measures captured the essential information content for the MHO phenotype, there were differences between studies in the way that specific variables such as blood pressure and serum lipid levels were measured. Also, our cut-off values for non-fasting measurements of, for example, blood glucose may underestimate the actual degree of the MHO present in the corresponding studies. Third, although many participating cohort studies included several thousands of participants, their health and lifestyle habits may not always be representative of the general population in this specific country because of bias in participation or differences in recruitment of participants. We also cannot exclude that a potential participation bias could affect the results [54]. As such, higher participation rates from either healthy or unhealthy individuals can influence the outcome, and it cannot be ruled out that the high percentage of MHO in the LifeLines Cohort Study may – at least in part – be explained by a preponderance of healthy individuals willing to participate.

An important factor to discuss is the time period in which the initial screening of each individual cohort was performed. Data in some cohorts were collected in the 1990s, while, for example, the participants in the Dutch LifeLines Cohort Study were recruited between 2007 and 2012, and in the Italian CHRIS study after August 2011. There have been several changes in environmental factors such as health behaviour and smoking pattern over time, which may have a bearing on the prevalence of MetS and on health in general. In many countries higher awareness of the importance of increased physical activity [55] or smoking cessation [56,57] have been recognized, although it appears that the current epidemic of obesity is still on-going [2]. As an example, cessation of smoking is on one hand associated with weight gain [58], which may be perceived negatively by individuals [59], but it also results in improvement of the metabolic profile as smoking cessation is accompanied by an increase of HDL cholesterol and reduction of triglycerides [60]. It is important to note that the major objective of this descriptive study was to evaluate the phenomenon of healthy obesity among the participating European population-based studies. The BioSHaRE-HOP consortium is currently expanding its harmonization efforts, and assessing differences in lifestyle factors such as nutritional habits, physical activity, smoking and general awareness of health between the various participating countries in order to have a better estimate of the characterization and the determinants of (healthy) obesity.

## Conclusion

In summary, we report the first scientific results of this collaborative project on the prevalence of healthy obesity within a FP7 funded consortium, BioSHaRE-EU. We have co-analysed data across the participating studies by applying careful harmonization algorithms. The present findings indicate considerable variation in the occurrence of MHO across the different European populations even when unified criteria or definitions were used to classify this phenotype. Further studies are needed to identify the underlying factors for these differences. This area of research will improve our understanding of obesity in general and possibly identify novel preventive measures for the consequences of obesity.

## Competing interests

The authors declare that they have no competing interests.

## Authors’ contributions

MP, BHRW, RPS, PB, IF conceived the study. JVvVO, MLN, SNS, DD, MP and BHRW have been involved in integration of all analyses, data interpretation and drafting the manuscript. MLN, JVvVO, SNS, KF, LF, AJ, CN, CP, HLH, ER, KK coordinated and performed the harmonization and local analysis and interpretation of the data of all participating studies. AG, MG, MH, TH, EP, CS, MLT, MAS, PB, VF, IF, LG, JH, JEKR, TLH, SM, AM, KM, MJM, AP, PPP, TS, VS, RPS, MU, PvdH, MMvdK, MW, MP and BHRW were involved in local study design, collection of data, and/or coordination and execution of measurements and biochemical analyses. LG, JRH helped with the data interpretation and drafting the manuscript. All authors provided intellectual contributions to the manuscript and have read and approved the final version.

## Authors’ information

Jana V van Vliet-Ostaptchouk, Marja-Liisa Nuotio, Sandra N Slagter, Dany Doiron, equal contribution as first author; Markus Perola, Bruce HR Wolffenbuttel, equal contributors as last author.

## Pre-publication history

The pre-publication history for this paper can be accessed here:

http://www.biomedcentral.com/1472-6823/14/9/prepub

## Supplementary Material

Additional file 1 **Study descriptions and methodologies.** See enclosed file with supplementary information. Click here for file
