# Supplementary material for: The prevalence of metabolic syndrome and metabolically healthy obesity in Europe: a collaborative analysis of ten large cohort studies
Source: BMC Endocr Disord. 2014 Feb 1;14:9. doi: 10.1186/1472-6823-14-9 (PMC3923238; doi:10.1186/1472-6823-14-9)
Supplement: Additional file 1 — Study descriptions and methodologies. See enclosed file with supplementary information. [file 1472-6823-14-9-S1.pdf]

The prevalence of Metabolic syndrome and Metabolically Healthy Obesity in Europe: a collaborative analysis of ten large cohort studies.

Supplementary material

Study descriptions and methodologies

Estonia (EGCUT)

The Estonian cohort is from the population-based biobank of the Estonian Genome Project of University of Tartu (EGCUT). The current cohort size is over 51,515 individuals, from 18 years of age and up, which reflects broadly the age distribution in the adult Estonian population. Subjects were recruited by their general practitioners (GP) or especially trained recruiters (at the recruitment centers managed by EGCUT), on voluntary basis. Each participant filled out a Computer Assisted Personal interview during 1-2 hours, which included questions related to personal data (place of birth, place(s) of living, nationality etc.), genealogical data (family history, three generations), educational and occupational history and lifestyle data (physical activity, dietary habits, smoking, alcohol consumption, women's health, quality of life). Anthropometric and physiological measurements were taken by trained personnel. Several anthropometric parameters were measured during the recruitment interview: height, weight, waist and hip circumference. Blood pressure measurement was not standardized – a single measurement was taken using a conventional method (a mercury manometer or a digital manometer. A venous blood sample was obtained from each participant. The DNA, plasma, and white blood cells were immediately isolated, packaged into the CBSTM High Security straws, and are stored in liquid nitrogen. The blood samples were obtained without prior fasting, although time since last meal was recorded in the questionnaire. Total cholesterol, HDL-cholesterol, triglycerides and blood glucose were measured using the NMR (Nuclear Magnetic Resonance) technique on stored plasma samples, at Mika-Ala Korpela's lab in Oulu, Finland.
In this study, the medical history and current health status of the participants are recorded according to the International Classification of Diseases, 10th revision (ICD-10). This includes a complete list of all diseases for each participant based on the information provided by the

participant or retrieved from the health records in hospital databases, national registries and National Digital Health Records.

The data represented in this manuscript have been collected between 2002 and 2010 (for 92.2% of the participants this was between 2007 and 2010).

Finland

The National FINRISK 2007 study (DILGOM)

In Finland, cardiovascular risk factors have been studied with population-based health surveys at five year-intervals since 1972 and are called The National FINRISK Study [1]. These studies are used for examining trends in national health as well as for research purposes. The DILGOM sample is an extension of the National FINRISK Study 2007 survey that consists of Finnish individuals of 25-74 years age who participated in the DIetary, Lifestyle and Genetic determinants of Obesity and Metabolic syndrome study which was carried out in Helsinki/Vantaa region in Southern Finland. All of the participants were aged 25–74 years and white Caucasians.

Blood pressure was measured with the mercury manometer in sitting position after at least 5 minutes of rest. Cuff of the manometer was 14 cm x 40 cm of size and measurements were taken from the right arm of the individual. Systolic blood pressure was determined using Korotkoff I as a point of definition and diastolic blood pressure using Korotkoff V as a point of definition. Measurement was repeated three times with the intermission of one minute in between of measurements. The variables of systolic and diastolic blood pressure were defined to be the average of the 2nd and 3rd measurement. Anthropometric measurements of weight and height were obtained using standard measure and scale. Waist circumference was measured at a level midway between the lower rib margin and iliac crest with the tape all around the body in standing position. Hip circumference was measured at the level of widest circumference over the greater trochanters. All of the measurements were performed by trained study nurses. Information about medical history and medication of a participant were received from a specific questionnaire. A confirmed clinical diagnosis for stroke, myocardial infarction or cardiac failure at baseline were used for identification of the presence of CVD.

Blood sampling was performed in the fasting state, by choice after at least 10h duration of fast. Blood samples were left at room temperature for 45 min before the serum and plasma were separated via centrifugation. Total cholesterol was measured with an enzymatic colorimetric method, HDL-cholesterol with a direct homogenous enzymatic colorimetric determination (CE-CO-POD method), and triglycerides with an enzymatic colorimetric determination (GPO-PAP method). Fasting blood glucose was measured with a glucose hexokinase method. All samples were stored in a freezer at -80°C. Measurements were performed 2-3 months after sampling.

The data represented in this manuscript have been collected in the year 2007.

Finland (Health 2000)

Health 2000 is a population based national health survey about the health and functional capacity of Finnish individuals (http://www.terveys2000.fi/julkaisut/baseline.pdf). The main aim of the survey is to study the prevalence and determinants of the most important health problems and the associated need for care, rehabilitation and help among the working-aged and aged population. A nationally representative sample of 10 000 individuals has been drawn of the population aged 18 and over. The results of this study are used for examining trends in national health as well as for research purposes. All of the participants were white Caucasians.

Blood pressure was measured with the mercury manometer (Mercuro 300) in sitting position after at least 5 minutes of rest. Cuff of the manometer was 12 cm x 35 cm or 15 cm x 43 cm of size and measurements were taken from the right arm of the individual. Systolic blood pressure was determined using Korotkoff I as a point of definition and diastolic blood pressure using Korotkoff V as a point of definition. Measurement was repeated two times with the intermission of two minutes in between of measurements. The variables of systolic and diastolic blood pressure were defined to be an average of 1st and 2nd measurement. Anthropometric measurements of weight and height were obtained using standard measure and scale (Person-Check, Medizintechnik, KaWe, Kirchner & Wilhelm, Germany). Waist circumference was measured at a level midway between the lower rib margin and iliac crest with the tape all around the body in standing position. Hip circumference was measured as the maximal circumference in standing position. All of the measurements were performed by trained technicians. When forming the variable defining the presence of CVD in Health2000, three separate data points were combined. Confirmed clinical diagnosis for stroke at baseline, confirmed clinical diagnosis for myocardial infarction at baseline and confirmed clinical diagnosis for cardiac failure at baseline were combined to obtain one variable, prevalent CVD at baseline which was used in analyses.

Blood sampling was performed in the fasting state, after at least four hours of fasting. Total cholesterol was measured with an enzymatic colorimetric method, HDL-cholesterol with a direct homogenous enzymatic colorimetric determination (CE-CO-POD method), and triglycerides with an enzymatic colorimetric determination (GPO-PAP method). Fasting blood glucose was measured with a glucose hexokinase method. All samples were stored in freezer at -70°C. Measurements were performed 2-3 months after sampling.

The data represented in this manuscript have been collected in the year 2000.

Germany (KORA)

The Cooperative Health Research in the Region of Augsburg (KORA) study is a series of independent population-based epidemiological surveys and follow-up studies of participants living in the region of Augsburg, Southern Germany [2]. The present study is based on data of the follow-up study KORA F4 (2006/2008) of the KORA S4 survey (1999/2001). History of CVD was defined as self-reported myocardial infarction, stroke and/or angina pectoris. All participants of KORA F4 are 32-82 years of age, and are of German white Caucasian origin. Body weight was measured in light clothing by trained investigators to the nearest 0.1 kg, and height to the nearest 0.5 cm. Waist circumference was measured at the minimum abdominal girth and hip circumference was assessed at the maximum protusion of the hips at the level of the symphysis pubis to the nearest 0.1 cm [3].

An oscillometric digital blood pressure monitor (HEM-705CP, Omron Corporation, Tokyo, Japan) was used for blood pressure (BP) measurement. Three BP recordings were taken at the right arm of seated subjects after at least 5 min at rest. The pause between readings was 3 min. The mean of the 2nd and 3rd measurement was used for the present analyses.

The blood samples were drawn in the morning between 8:00 a.m. and 10:00 a.m. after a period of overnight fasting into serum gel tubes, gently inverted two times and then allowed to rest for 30 min at room temperature (18−25°C) to obtain complete coagulation. The material was then centrifuged at 15°C for 10 min at 2750*g*. All analyses were performed within a maximum of 6 h after withdrawal. All lipids were measured using the Dimension RxL (Dade Behring). Total cholesterol was determined by cholesterol esterase method (CHOL Flex, Dade-Behring, CHOD-PAP method), HDL-cholesterol was measured using the AHDL Flex (Dade-Behring, CHOD-PAP method after selective release of HDL) and triglycerides were measured using a TGL Flex (Dade Behring, enzymatic colorimetric test, GPO-PAP method). Blood glucose was analysed using a hexokinase method (Gluco-quant; Roche Diagnostics, Mannheim, Germany).

The data represented in this manuscript have been collected in the year 2006-2008.

Italy (CHRIS)

The CHRIS project (Collaborative Health Research in South Tyrol Study) is a longitudinal population-based study centered around the health of people living in Val Venosta, South Tyrol, Italy. The main objective of the study is to identify genetic and environmental effects, as well as gene-environment interaction effects, on a number of common disease outcomes and intermediate phenotypes. Also, a long-term objective is to provide a long-term platform for population-based research in South Tyrol. Subjects are invited to participate by personal letter; if consented, they undergo a core assessment protocol consisting of at least interview, blood drawing, urine sampling, anthropometry, ECG and blood pressure, for an approximate

duration of three hours. The CHRIS Study has started in August 2011 and participants' recruitment will continue for five years; currently 1117 individuals have been included in this analysis and all of them are of Western European descent. Inclusion age of participants was 18 years and above.

For anthropometric measurements, all participants were wearing light clothes and were barefoot. The weight measurement was performed with a OMRON BF508 Body Composition Monitor with an accuracy of 0.1 kg. The body height was read to the nearest 0.1 cm on a body height scale. All measurements were taken once by trained nurses or study assistants.

Blood pressure and pulse rate were measured three times after a twenty-minute rest, using the digital automatic blood pressure monitor OMRON M10-IT. Measurements were done according to the instrument manufacturer's guidelines. The three blood pressure and pulse measurements were automatically taken by the device once every two minutes and their averaged result was immediately stored in a database. During the measurements, the participant did not talk or move and sat upright with his/her back straight, legs not crossed and body relaxed. The arm was placed on a table so that the cuff was at the same level of the heart.

Information about medical history (e.g. cardiovascular diseases and diabetes) and medication of a participant were received from an interviewer-administered questionnaire. History of cardiovascular disease was defined as positive if at least one of the following conditions was present: angina, myocardial infarction, bypass implantation, coronaropathy, syncope, atrial fibrillation, stroke, transitory ischemic attack (TIA), heart failure, pulmonary edema.

Blood was taken after a 12h overnight fast bwteeen 8 and 10 a.m. Levels of lipids and glucose were measured on fresh samples using the Hitachi Modular P machine; assays used were Cobas® Gluco-quant Glucose/HK (glucose), Cobas® HDL-C plus 3rd generation (HDL), Cobas ® Triglyceride GPO-PAP (triglyceride) and Cobas® Cholesterin CHOD-PAP (total cholesterol).

The data represented in this manuscript have been collected between August 2011 and November 2012.

Italy (MICROS)

The MICROS study is a population-based survey carried out on Stelvio, Vallelunga and Martello, three Alpine villages of the Val Venosta, South Tyrol, Italy [4]. The small villages are characterized by old settlement, small number of founders, high endogamy rates and slow/null population expansion. Participants were invited by public advertisement and recruited in 2002-2003. A standardized, interviewer-administered questionnaire was administered to the participants and clinical measurements, blood and urine samples were collected. Also, comprehensive genealogies spanning 15 generations were reconstructed.

Inclusion age of participants was 18 years and above. A total number of 1060 individuals have been included in this data analysis and all of them are white Caucasians.

For anthropometric measurements, all participants were wearing light clothes and were barefoot. The weight measurement was performed with an accuracy of 0.1 kg. The body height was read to the nearest 0.5 cm on a body height scale. All measurements were taken once by trained nurses or study assistants.

Blood pressure and pulse rates were measured three times after a three-minute rest, using a blood pressure monitor HEM-705CP (OMRON). During the measurements, the participant did not talk or move and sat upright with his/her back straight, legs not crossed and body relaxed. The arm was placed on a table so that the cuff was at the same level of the heart. The averaged result of the three measurements was used to prepare the tables of this paper.

Information about medical history and medication of a participant were received from interviewer-administered questionnaire. History of cardiovascular disease was defined as positive if at least one of the following conditions was present: angina, myocardial infarction, stroke, transitory ischemic attack (TIA), pulmonary edema.

Blood sample was taken after a 12h overnight fast. Blood samples were prepared and stored in a -80 ºC freezer. Levels of glucose were measured using the hexokinase assay methodology on a Dimension RxL automated analyze (Dade Behring Newark, DE, USA). Levels of lipid were measured using the ADVIA®1650 machine (Siemens Healthcare Diagnostics); the assay methodology applied was enzymatic colorimetric determination (CE-CO-POD method) for total cholesterol determination, selective catalase elimination followed by enzymatic colorimetric determination (CE-CO-POD method) for HDL-cholesterol, and enzymatic colorimetric method (LIP-GK-GPO-POD) for triglycerides.

The data represented in this manuscript have been collected between September 2002 to December 2003.

The Netherlands (LifeLines)

The LifeLines Cohort Study is a multi-disciplinary prospective population-based cohort study examining in a unique three-generation design the health and health-related behaviors of 165,000 persons living in the North East region of The Netherlands [5]. It employs a broad range of investigative procedures in assessing the biomedical, socio-demographic, behavioral, physical and psychological factors which contribute to the health and disease of the general population, with a special focus on multimorbidity. In addition, the LifeLines project comprises a number of cross-sectional sub-studies which investigate specific age-related conditions. These include investigations into metabolic and hormonal diseases, including obesity, cardiovascular and renal diseases, pulmonary diseases and allergy, cognitive function and depression, and musculoskeletal conditions. All survey participants in the present

manuscript were between 18 and 80 years old at the time of enrollment, and of Western European descent. Participants were recruited through their general practitioner. Recruitment has been going on since the end of 2006, and until February 2013 over 120,000 participants have been included. For more information, see www.lifelines.net.

At the first visit, a number of physical investigations were conducted by trained research nurses. Height was measured to the nearest centimeter (cm) and weight to the nearest 0.5 kilogram (kg). Waist circumference was measured at a level midway between the lower rib margin and iliac crest with the tape all around the body in horizontal position. Hip circumference was measured as the maximal circumference over the buttocks. Systolic and diastolic blood pressure, and pulse rate were measured every minute for a period of 10 minutes using an automated DINAMAP Monitor i.e. 10 measures for each of the indices. The size of the cuff was chosen according to the arm circumference. The reported level for each of the blood pressure indices is the mean of the last 3 measures. History of CVD was defined as self-reported myocardial infarction, stroke, angina pectoris, or coronary artery bypass grafting.

Blood sampling was performed during the second visit, in the fasting state, between 8 and 10 am. The samples were then transported in temperature-controlled conditions (at room temperature or at 4 °C depending on the sample requirements) to the LifeLines laboratory facility. Measurements were carried out on fresh samples. Total cholesterol was measured with an enzymatic colorimetric method (CHOD-PAP), HDL-cholesterol with a direct enzymatic colorimetric method (PEG-modified cholesterol esterase/cholesterol oxidase), triglycerides with an enzymatic colorimetric method (GPO-PAP) and fasting blood glucose with a hexokinase method on a Roche Modular P chemistry analyser.

The data represented in this manuscript have been collected between December 2006 and January 2012.

The Netherlands (PREVEND)

For the PREVEND study (Prevention of REnal and Vascular ENd stage Disease), running in the city of Groningen, the Netherlands, all inhabitants of the city of Groningen between the ages of 28 and 75 years (85,421 subjects) were asked to send in a morning urine sample and to fill out a short questionnaire on demographics and cardiovascular history [6]. A total of 40,856 subjects (47.8%) responded. From this group, 30,890 subjects had a urinary albumin concentration of <10 mg/L and 9,966 subjects had a urinary albumin concentration of >10 mg/L in their morning urine sample. After exclusion of subjects with type 1 diabetes and pregnant women, all subjects with a urinary albumin concentration of >10 mg/L (n=7,768) together with a randomly selected control group with a urinary albumin concentration of <10 mg/L (n=3,395) were invited for further investigations in an outpatient clinic (total

n=11,163). A total of 8,592 subjects completed the total screening program, which consisted of two visits.

Data on demographic characteristics, co-morbid conditions, family history of diabetes, and the use of medication for diabetes, hypertension, or hyperlipidaemia were obtained during the baseline visit. Demographic information included age, gender, and self-reported race. History of cardiovascular disease was collected using a self-administered questionnaire that was validated during baseline visit. It was based on an evaluation of the history of heart attacks, heart surgery, angioplasty and (ischaemic stroke, haemorrhagic) stroke, but not ontransient ischemic attack.

For anthropometric measurements, all participants were wearing light clothes and were barefoot. Weight was measured to the nearest 0.5 kg with a Seca balance scale (Vogel and Halke, Hamburg, Germany). Height was measured to the nearest 0.5 cm. Minimal waist circumference was measured on bare skin at the natural indentation between the 10th rib and the iliac crest. At both visits, blood pressure was measured in supine position, every minute, for 10 and 8 min, respectively, with an automatic Dinamap XL Model 9300 series device (Johnson and Johnson, Medical Inc., Arlington, TX). Systolic and diastolic BP were calculated as the mean of the last two measurements of the two visits.

Blood samples were taken after a 12h overnight fast, after 15 minutes of rest. Plasma glucose was measured shortly after blood sampling. Plasma was obtained by centrifugation at 4ºC, and the samples were stored at -80 ’C until analysis. Plasma glucose and total cholesterol were assessed using Kodak Ektachem dry chemistry (Eastman Kodak, Rochester, New York, USA). HDL cholesterol was measured with a homogeneous method (direct HDL, AEROSETTM System, Abbott Laboratories, Abbott Park, USA). An enzymatic colorimetric determination GPO-PAP method was used for determining triglyceride levels.

The data represented in this manuscript have been collected between September 1997 and November 1998.

Norway (HUNT2)

The Nord-Trøndelag health study (HUNT) is one of the largest health studies ever performed comprising the population of the county of Nord-Trøndelag in the middle part of Norway [7]. It is a unique database of personal and family medical histories collected during three intensive surveys. The adult population included in the surveys comprise: HUNT1 (1984-1986, age ≥ 20 years), n= 77,212 (89.4% participation rate), HUNT2 (1995-1997, age ≥ 20 years), n=65,237 (69.5% participation), and HUNT3 (2006-2008, age ≥ 20 years), n=50,807 (54.1% participation). Data collection included self-reported questionnaires, structured interviews, clinical measurements. The HUNT study is reinforced and supplemented by cross referencing with registries at the regional level (registries such as radial and hip fractures,

venous thrombosis, pulmonary embolism, ischaemic heart disease and stroke) and with registries at the national level (The Cancer Register, The Medical Birth Register, and The National Health Insurance Register). Additionally, Statistics Norway provides necessary information from The Population Census Register and The Family Register to create a genealogical database ("family trees"). More details can be found on the website **http://www.ntnu.no/hunt.** In 2000, 97% of the Nord-Trøndelag population was shown to be of Caucasian origin and the percentage of immigrants from other ethnic groups was even lower before that. There is, however, a small Lappish (Sami) population in Nord-Trøndelag. The participants from the HUNT2 study were included in the present study.

For anthropometric measurements the participants wore light clothing and were barefoot. Height was measured to the nearest centimeter (cm) and weight to the nearest 0.5 kilogram (kg). Waist- and hip circumference were measured to the nearest centimeter applying non-stretchable band horizontally; waist circumference at the umbilical level after the participants emptied their lungs, or midway between the last rib and the iliac cristae if the latter was larger and hip circumference at the widest part of the gluteal muscle.

Blood pressure and heart rate were measured by specially trained nurses or technicians using a Critikon Dinamap 845XT based on oscillometry. Cuff size was adjusted after measuring the arm circumference. The measurement started after the participant had been seated for two minutes with the cuff on the arm, and the arm resting on a table. Blood pressure and heart rate were measured automatically three times at one minute intervals. Blood pressures measured with the Dinamap device are slightly lower than those measured with a sphygmomanometer, especially for diastolic blood pressure.

In HUNT2 the definition for the history of CVD or (medical treatment for) diabetes or hypertension was questionnaire based (Have you had or do you have myocardial infarction (heart attack), angina pectoris (chest pain), stroke/brain haemorrhage or diabetes? Are you taking medication for high blood pressure?).

Blood was drawn in the non-fasting state. Measurement of blood glucose and lipids were carried out on fresh blood samples using Hitachi 911 Autoanalyzer. Glucose was measured with a glucose hexokinase method. Total cholesterol was measured with an enzymatic colorimetric cholesterol esterase method, HDL-cholesterol with an enzymatic colorimetric cholesterol esterase method after precipitation with phosphor tungsten and magnesium ions, and triglycerides with an enzymatic method.

Only HUNT2 data collected between the years 1995 and 1997 have been included in this manuscript.

United Kingdom (NCDS)

The 1958 birth cohort or the National Child Development Study (NCDS) began as a study of Perinatal Mortality focussing on just over 17,000 births in a single week in 1958. To address concerns regarding the stillbirth rate not falling, the original study aimed to identify social and obstetric factors linked to stillbirth and neonatal death. The initial survey was not planned as a longitudinal study, but subsequently participants have been followed up at different intervals resulting in a range of variables being recorded at different times. One of these recording in 2002 involved blood being taken from participants resulting in the derivement of biological and genomic data making the 1958 birth cohort into a biobank [8]. The study is from the 2002 sweep of the 1958 birth cohort (N=9,377), with complete data for variables requested (N=7,346). All subjects were 44 years old at times measurements were taken. The majority of participants in NCDS were of European origin.

All anthropometric measurements were carried by a nurse using the standard protocol. Standing height was measured to the nearest cm without shoes. Weight was measured to the nearest kg if the participant was over 150 kg then weight was not recorded. Weight and hip circumference was measured to the nearest mm. If any anthropometric measurements were deemed unreliable by the nurse recording these, then this was also recorded. Blood pressure was taken with an Omron 907 blood pressure monitor, with standard and large adult cuffs. Blood pressure and heart rate were measured automatically three times at one minute intervals after rest. Blood pressure and heart rate were taken to be the mean of all three measurements.

Venous blood samples were obtained without prior fasting; participants could choose whether to sit or lie down when blood was taken. Plasma glucose levels were not measured, but for the purpose of this study derived from HbA1c based on the regression equation between HbA1c and fasting blood glucose calculated in the participants of the LifeLines Cohort Study. Glycosylated haemoglobin (HbA1c) was measured on whole citrated blood by ion exchange high performance liquid chromatography, using the Tosoh A1c 2.2 Glycohemoglobin Analyser HLC-723GHb. Serum triglycerides, and total and HDL- cholesterol were measured in serum by Olympus model AU640 autoanalyser in a central lab in Newcastle. Enzymatic colorimetric determination GPO-PAP method was used to determine triglycerides, CHOD-PAP method for total cholesterol and for HDL-cholesterol. Blood samples were stored in a -80 ^{o}C freezer.

In NCDS, the ICD-10 codes for myocardial infarction, stable ischaemic heart disease and unstable ischaemic heart disease were used to define the history of CVD. The ICD10 codes were reported by a research nurse in communication with the participants during a telephone interview.

Participants in the 2002 study were asked about current medication use, and all medications were coded according to the British National Formulary (BNF). Therefore, we used the BNF codes for diabetes, blood pressure and CVD.

The definition of cardiovascular disease

The criteria for the definition of CVD varied slightly between cohort studies. However, in general assessment proved to be very well comparable. In EGCUT the identification of the participants with cardiovascular diseases was based on ICD-10 codes (I10-I79), as well as in NCDS where the ICD10 codes for myocardial infarction, stable ischaemic heart disease and unstable ischaemic heart disease were used. For about 50% of participants from the EGCUT study, the disease data is obtained directly from medical records, the remainder being based on self-reported data. For NCDS, this was self-reported; data were collected during a telephone interview. In the Finnish cohorts DILGOM and Health 2000, self-reported diagnosis for stroke, myocardial infarction or cardiac failure at baseline were used for identification of the presence of CVD. In KORA participants with a history of myocardial infarction, stroke or angina pectoris were excluded from analysis. In the Italian CHRIS cohort, the history of cardiovascular disease was defined as positive if at least one of the following conditions was present: angina pectoris, myocardial infarction, bypass implantation, coronaropathy, syncope, atrial fibrillation, stroke, transitory ischemic attack (TIA), heart failure, pulmonary edema. In MICROS the history of cardiovascular disease was defined as positive if at least one of the following conditions was present: angina, myocardial infarction, stroke, transitory ischemic attack (TIA), pulmonary edema. For both CHRIS and MICROS, information was received from an interviewer-administered questionnaire. To define the status of CVD in individuals from LifeLines, the history for prevalent diabetes, coronary heart disease, congestive heart failure, peripheral vascular disease, as well as previous stroke or transient ischemic attack was self-reported and derived from standardized questionnaires. In the PREVEND study, history of cardiovascular disease was collected using a self-administered questionnaire that was validated during baseline visit. It was based on an evaluation of a history of heart attacks, heart surgery, angioplasty and (ischaemic or haemorrhagic) stroke, but not on transient ischaemic attacks. Also, in HUNT2 the definition was questionnaire-based (Have you had or do you have myocardial infarction (heart attack), angina pectoris (chest pain) or stroke/brain haemorrhage?).

References

1. Inouye M, Kettunen J, Soininen P, Silander K, Ripatti S, Kumpula LS *et al.*: **Metabonomic, transcriptomic, and genomic variation of a population cohort.** *Mol Syst Biol* 2010, **6:** 441.

2. Wichmann HE, Gieger C, Illig T: **KORA-gen--resource for population genetics, controls and a broad spectrum of disease phenotypes.** *Gesundheitswesen* 2005, **67 Suppl 1:** S26-S30.

3. Liese AD, Doring A, Hense HW, Keil U: **Five year changes in waist circumference, body mass index and obesity in Augsburg, Germany.** *Eur J Nutr* 2001, **40:** 282-288.

4. Pattaro C, Marroni F, Riegler A, Mascalzoni D, Pichler I, Volpato CB *et al.*: **The genetic study of three population microisolates in South Tyrol (MICROS): study design and epidemiological perspectives.** *BMC Med Genet* 2007, **8:** 29.

5. Stolk RP, Rosmalen JG, Postma DS, de Boer RA, Navis G, Slaets JP *et al.*: **Universal risk factors for multifactorial diseases: LifeLines: a three-generation population-based study.** *Eur J Epidemiol* 2008, **23:** 67-74.

6. Pinto-Sietsma SJ, Janssen WM, Hillege HL, Navis G, de ZD, de Jong PE: **Urinary albumin excretion is associated with renal functional abnormalities in a nondiabetic population.** *J Am Soc Nephrol* 2000, **11:** 1882-1888.

7. Krokstad S, Langhammer A, Hveem K, Holmen T, Midthjell K, Stene T *et al.*: **Cohort Profile: The HUNT Study, Norway.** *Int J Epidemiol* 2012 Aug 9 (online).

8. Power C, Elliott J: **Cohort profile: 1958 British birth cohort (National Child Development Study).** *Int J Epidemiol* 2006, **35:** 34-41.
